# Cardiovascular Disease in Systemic Lupus Erythematosus: The Role of Traditional and Lupus Related Risk Factors

**DOI:** 10.2174/157340308784245775

**Published:** 2008-05

**Authors:** Carlos Borelli Zeller, Simone Appenzeller

**Affiliations:** 1 Instituto Dante Pazzanese de Cardiologia; 2 Rheumatology Unit, Department of Internal Mdicine-State University of Campinas, Brazil and McGill University, Canada

**Keywords:** Cardiovascular disease, systemic lupus erythematosus, atherosclerosis.

## Abstract

Atherosclerosis is a chronic inflammatory disorder characterized by immune cell activation, inflammation driven plaque formation and subsequent destabilization. In other disorders of an inflammatory nature, the chronic inflammatory state per se has been linked to acceleration of the atherosclerotic process which is underlined by an increased incidence of cardiovascular disease (CVD) in disorders such as systemic lupus erythematosus (SLE), rheumatoid arthritis (RA) and antiphopholipid (Hughes) syndrome (APS). SLE is an autoimmune disease that may affect any organ. Premature coronary heart disease has emerged as a major cause of morbidity and mortality in SLE. In addition to mortality, cardiovascular morbidity is also markedly increased in these patients, compared with the general population. The increased cardiovascular risk can be explained only partially by an increased prevalence of classical risk factors for cardiovascular disease; it also appears to be related to inflammation. Inflammation is increasingly being considered central to the pathogenesis of atherosclerosis and an important risk factor for vascular disease. Recent epidemiologic and pathogenesis studies have suggested a great deal in common between the pathogenesis of prototypic autoimmune disease such as SLE and that of atherosclerosis.

We will review traditional risk factors for CVD in SLE. We will also discuss the role of inflammation in atherosclerosis, as well as possible treatment strategies in these patients.

## INTRODUCTION

Systemic lupus erythematosus (SLE) is an autoimmune disease that primarily affects young women. Cardiac involvement in patients with SLE can involve all components of the heart, including the pericardium, conduction system, myocardium, valves, and coronary arteries [1, 2]. Coronary artery disease (CAD) in SLE was described much later than the other cardiovascular manifestations. The clinical manifestations of CAD in SLE can result from several pathophysiologic mechanisms, including atherosclerosis, arteritis, thrombosis, embolization, spasm, and abnormal coronary flow [2].

The striking clinical characteristic of most patients with SLE who have a myocardial infarction is their young age. This demographic characteristic suggests that patients with SLE are at increased risk of myocardial infarction and that reports of myocardial infarction in patients with SLE do not simply represent chance occurrences. Fatal myocardial infarction has been reported to be 3 times higher in patients with SLE than in age- and gender -matched control subjects [1, 4]. Recent case- control series have confirmed that the risk of myocardial infarction in patients with SLE is increased between 9- and 50-fold over that in the general population [1-3]. It has been increasingly recognized that patients with SLE have a high cardiovascular mortality. Lupus is now considered to be an independent risk factor for the development of atherosclerosis. Viewing atherosclerosis as an inflammatory disease, this association becomes stronger and better understood.

Epidemiological observations have linked inflammation with the cardiovascular events [1, 2]. Clinical epidemiological observations strongly suggest that, together with classical conventional risk factors, other mechanisms (non-conventional/disease-specific factors) promote accelerated atherosclerosis in diseases like SLE and other rheumatic diseases [3-7]. The excess risk observed in autoimmune disease appears to be driven by systemic inflammation, directly or indirectly through its damaging effects on the vasculature; and thus the concept of inflammation as a cardiovascular risk factor [3, 6, 7].

We will review the association of atherosclerosis and inflammation in general population, with emphasize in the role of endothelial dysfunction and autoantibodies. We further will review the presence of traditional and nontraditional risk factors in SLE patients.

## ATHEROSCLEROSIS AND INFLAMMATION

Inflammation and atherosclerosis have been linked for decades, although the underlying mechanism and the antigens causing immune activation are not totally elucidated [7-10]. Activated monocytes, macrophages and T cells and cytokines have an important role in atherogenisis. C reactive protein (CRP) is considered an indentpendent risk factor for CVD. In addition, autoantigens and autoantibodies have also important roll in atherogenisis in general population [7, 10].

### Endothelium and Atherosclerosis

The endothelium is a single layer of cells that lines the luminal surface of blood vessels [8-10]. It is strategically situated to act as a direct interface between the components of circulating blood and local tissue. It regulates numerous local blood vessel functions, including vascular tone, cell adhesiveness, coagulation, inflammation, and permeability. The endothelium is able to produce and react to several potent, locally active mediators and it recruits inflammatory cells to sites of tissue damage [9-10]. Endothelial dysfunction is a key event in atherogenesis and appears before the histopatholical evidence of atherosclerotic lesion [7, 11]. Chronically raised levels of inflammatory mediators may drive the inflammation that subsequently contributes to endothelial damage [12]. Chronic ED and vascular inflammation may be induced both by conventional risk factors and systemic inflammation are important mechanisms in atherogenesis [11, 12].

During the initial stages of atherosclerosis, monocytes and T cells are recruited to the vessel wall across an intact endothelium. In order to pass the endothelium, adhesion molecules are necessary to be expressed in the inner layer of the endothelium. Several adhesion molecules may promote the adherence of monocytes, including intercellular adhesion molecule 1[ICAM-1], vascular cell adhesion molecule 1 [VCAM-1] and selectins) [7, 10]. These adhesion molecules are surface proteins and their expression are mainly regulated by transcription [10, 13] and induced by pro-inflammatory mediators, such as tumour necrosis factor alpha (TNF-α), interleukin-1 (IL-1), C-reactive protein (CRP) and CD40/CD40 ligand interaction [7, 10-12, 14-16].

### Inflamation and Atherosclerosis in General Population

Systemic inflammation may be regarded as accelerating the atherosclerotic process, in especially CRP has been associated with CVD risk in the general population. CRP, an acute phase protein commonly used to measure inflammation in autoimmune diseases, has also an important role in atherogenisis of normal population, both in the early initiation of atherosclerotic lesions and in the conversion of stable to unstable plaques. The biological effect of CRP on atherosclerosis development seems to encompass a complex network of interactions with the cells involved in plaque growth and development. CRP has also proatherogenic properties by being capable of activating the complement system, inducing endothelial production of monocyte chemotactic protein-1(MCP-1) and secretion of endothelin-1 (ET-1) and interleukin 6 (IL-6), up regulating adhesion molecules (ICAM-1, VCAM-1, selectins), mediating macrophage uptake of LDL and stimulating monocyte production of tissue factor [7, 17-19].

Epidemiological and clinical studies have shown strong and consistent relationships between markers of inflammation and risk of future CVD events. Therefore CRP levels may be considered an independent risk factor for myocardial infarction and stroke in men with and without other traditional risk factors [17]. Prospective epidemiological studies have shown that increased levels of CRP, predicts coronary events in healthy individuals and in patients with stable and or unstable angina [20].

This strong predictive value of CRP may be explained by its long-term stability during storage, its long-life, its lack of diurnal variation, and its lack of age and sex dependency [7, 20].

### Autoantigens and Atherosclerosis

Evidence from studies of human disease supports the involvement of autoantigens in atherosclerosis. Numerous autoantigens and their respective autoantibodies are involved in the pathogenesis of atherosclerosis [21, 22]. A cellular immune response specifically directed against heat-shock proteins (HSPs), oxidized low-density lipoprotein (oxLDL), and _2-glycoprotein-I (_2GPI) has been reported, suggesting a direct involvement of these molecules in atherosclerosis [23, 24]. There are probably many more such specific cell lines reacting with specific antigens that can modulate atherosclerosis by either aggravating or decreasing its extent (proatherogenic or antiatherogenic).

### Heat-Shock Proteins (HSPs)

The human HSPs are a group of proteins that are involved in maintaining the folding of several cell components. They are expressed at high levels on endothelial cells in response to stress factors in order to maintain the viability of targeted cells and may therefore become a target for autoimmunity [21, 25]. Stress factors include hypertension, smoking, lipoproteins, oxidants and many others. There are clinical, subclinical, and experimental data that anti-HSP60/65 has a proatherogenic role. Patients with high levels of anti-HSP65 were found to be at increased risk of subsequent cardiovascular events and cardiovascular mortality [26, 27]. Furthermore, patients with sonographic evidence of carotid atherosclerotic lesions had significantly elevated levels of anti-HSP65 when compared to controls [22, 28].

### Anti-Oxidized Low-Density Lipoprotein (oxLDL)

LDL is constituted by molecules of phospholipids, triglycerides, and cholesterol, and accumulates in macrophages. During early atherogenesis, LDL become trapped in the subendothelial space and is subsequently oxidized [22, 29]. This oxLDL is thought to be responsible for triggering inflammatory responses in macrophages and vascular wall cells. It increases the adhesive properties of endothelial cells and induces the activation of monocytes and T cells [22, 30-32]. OxLDL is the type of LDL that is more likely to undergo uptake by macrophages, which turn into the foam cells characterizing atherosclerotic lesions. Anti-oxLDL antibodies are present in patients with atherosclerosis, independently of its etiology [33, 34]. Anti-oxLDL autoantibodies seems to be able to discriminate patients with peripheral vascular disease and control subjects and there is also a tendency for higher autoantibody levels in patients with more extensive atherosclerosis [33].

### Antiphospholipid Antibodies

Antiphospholipid antibodies are a heterogeneous group of autoantibodies, including, anticardiolipin antibody (aCL) and lupus anticoagulant (LA), generally directed to phospholipid binding proteins; in this regard, β2GPI represents the major antigenic target [21]. 2GPI can be found in human atherosclerotic lesions obtained from carotid endarterectomies, it is abundantly expressed within the subendothelial regions and the intimal-medial border of human atherosclerotic plaques, and it colocalizes with CD4_ lymphocytes [22, 35]. T cells specific for _2GPI are capable of increasing atherosclerosis, suggesting that _2GPI is a target autoantigen in atherosclerosis.

## SLE AND ATHEROSCLEROSIS

SLE is an autoimmune disease with a variety of clinical manifestations that occurs predominantly young women [22]. In the general population atherosclerosis is more frequently observed in the elderly [36] and young women are normally considered a group free from atherosclerosis, in part because of the protective effects of estrogen [22, 37].

The high incidence of atherosclerosis in young women with SLE was initially noted in autopsy studies by Bulkley and Roberts [38] in 1975 where significant extent of atherosclerosis was observed in more than 50% of deceased patients independently of the cause of death. This finding was emphasized in a report on myocardial infarction and sudden death in 1976 where Urowitz *et al.* [39] described a bimodal distribution of the causes of death in SLE: An “early” peak caused by SLE severity/activity or infections, and a “late” peak caused by CVD. Not only does atherosclerosis occur more frequently in SLE patients than in the general population, but there is also epidemiological and clinical evidence that it is accelerated in these patients [22, 40, 41].

The reason that premature coronary atherosclerosis develops in patients with SLE is unknown. The leading theory is that immune complex deposition causes the initial intimal damage, which is followed by accelerated development of atherosclerosis in patients with traditional risk factors [42].

Although the risk of CVD is raised in SLE, it is still not clear how atherosclerosis is related to this risk [43]. It should also be noted that SLE-related CVD and atherosclerosis may differ from other conditions such as diabetes and hypertension, where it is often assumed that these conditions confer an increased risk in general. It could be the case that CVD - like many other disease manifestations of SLE - only affects a subgroup of SLE patients [43]. Clearly, controlled, prospective studies are needed to establish whether CVD is a general feature of the disease or more a complication affecting only a subgroup of patients [43]. Several studies suggested that atherosclerosis in SLE patients is characterized by an increased risk of localized plaque but not increased atherosclerosis in general, as determined by IMT measurements, at least not in patients with less severe disease [43-47].

## CARDIOVASCULAR RISK FACTORS IN SLE

The prevalence of clinically manifest ischemic heart disease has ranged between 8% and 16% in various studies [48-51]. The frequency of subclinical coronary artery disease (CAD) is likely to be considerably higher. Perfusion abnormalities have been reported in up to 38% of adult SLE patients [52-54] and in 16% of children [55]. Through the use of various noninvasive methods, atherosclerosis was detected in 28-40% of SLE patients [56-60], and was associated with increasing age and longer disease duration [61-65].

One important issue in SLE patients is to establish which are the risk factors for CVD and the role o traditional and non-traditional risk factors [43] (Table **1**). Many SLE patients with renal disease or with a history of CVD are on treatment with blood pressure-lowering drugs, and in line with this, previous studies indicate that hypertension is an important risk factor for CVD in SLE [66]. In addition, smoking seems also to have an important role as traditional rosk factor in sle patients [67]. On the other hand treatment, especially the cumulative prednisone dose may also represent a non-traditional risk factor in these patients [68].

On important finding is to remember that in several studies, the Framingham risk factors did not fully account for CVD in SLE [69], therefore it is necessary to develop other methods to determine the subgroup of SLE patients that are at highest risk for CVD disease.

### Traditional Risk Factors

Only few studies have addressed the question of whether the frequency and level of traditional risk factors in SLE patients differ from those observed in age and sex-matched healthy controls [48, 69-72]. But there appear to be few significant differences between patients and controls, although significantly higher concentrations of plasma homocysteine and triglycerides were reported in SLE patients [70-72]. Some studies concluded that CAD in SLE patients was not associated with recognized risk factors for CVD [74-76]. This conclusion needs to be regarded with some caution since some significant differences in the level and/or frequency of these risk factors were detected in all studies comparing SLE patients with and without subclinical or clinical CAD [48].

Metabolic syndrome is considered an independent predictor of cardiovascular morbidity and mortality that identifies substantial additional cardiovascular risk beyond the sum of the individual risk factors. In the general population, men with the metabolic syndrome were 1.9 - 3.0 times more likely to die of any cause, and 2.9 - 4.2 times more likely to die from coronary heart disease. Women with the metabolic syndrome also had a 2-fold increased risk of major adverse cardiovascular events and death. In addition to the cardiovascular risk factors that comprise the metabolic syndrome, there is a strong relationship with inflammation. In patients with lupus an increased prevalence of the metabolic syndrome, was observed, suggesting a common mechanism between premature atherosclerosis and inflammation, both features of systemic lupus erythematosus [88].

Nonetheless, it is clear that traditional risk factors do not fully account for the high risk of accelerated atherosclerosis among SLE patients and this suggests that disease-related factors constitute an equal or even greater risk. In another study the authors analyzed risk factors associated with the development of plaques and concluded that risk factors associated with carotid plaque and IMT are those typically associated with cardiovascular disease in the general population, whereas the risk factors associated with vascular stiffness include SLE-specific variables related to immune dysregulation and complement metabolism [77]. Therefore two different mechanisms may account for CAD in SLE patients and multicentric observational studies are needed to identify the role of each risk factor.

### Non Traditional Risk Factors

Several nontraditional risk factors may be found in SLE patients, including renal disease and corticosteroid use. An association between renal disease and the prevalence of risk factors for CAD was reported in several studies [78-80].

Among a large variety of disease- or treatment-related factors, only the duration or the cumulative dose of prednisone was consistently found to predict either atherosclerosis and subclinical or clinically overt CAD [81-85], but CAD may also develop in patients that never received corticosteroids [85]. Furthermore, nephritis and corticosteroid therapy are known to aggravate hyperlipidemia, hypertension and obesity [48]. 

Other disease-related factors are the inflammatory mediators released as a result of the chronic systemic inflammation associated with SLE, immune complex-mediated endothelial cell damage, and antiphospholipid antibodies [48]. Increased levels of anti-oxLDL have been found in SLE patients [86-89], however the association with CVD is still unclear. The presence of antiphospholipid antibodies has been associated with angina and myocardial infarction [90-93]. Several studies have shown an association with increased carotid intima-media thickness or plaque [94-96]. In SLE patients, data on the serum concentration of anti-HSP65 antibodies are heterogeneous and inconclusive [86, 97]. 

The results available to date are too inconsistent to allow any definite conclusions as to the role of inflammatory mediators in premature atherosclerosis. It has been proposed that SLE should be considered an independent cardiovascular risk factor.

Unfortunately, the conflicting nature of the data available to date does not allow any conclusions about the pathophysiology of accelerated atherosclerosis in SLE patients or about possible preventive measures beyond the treatment of traditional risk factors. Prospective studies are necessary in order to address both of these issues [48].

## MANAGEMENT

Although there are multiple risk factors for premature atherosclerosis in SLE and the individual importance of each factor has not been determined, SLE patients have to be treated. Preventive and symptomatic treatment should follow internationally guidelines, there is evidence that rheumatologists are not adequately implementing preventive strategies [10, 42]. Considering that SLE patients are young premonopausal women, and therefore should be considered s a high risk population for atherosclerosis and CVD [10].

SLE patients who present with typical complaints should be worked up and managed as any other nonlupus patient [10]. Extra care should, however, be given to patients who present with nonspecific complaints such as chest discomfort, and/or dyspnea, and/or occasional palpitations [10].

Investigation with 99mTc-sestamibi myocardial perfusion SPECT is a useful noninvasive imaging modality to detect cardiovascular involvement in SLE patients with nonspecific clinical complaints of heart disease. In fact, this technique was able to detect perfusion abnormalities in 27 of 33 SLE patients with such complaints [98]. The presence of increased carotid IMT and the presence of discrete carotid plaque have been shown in many populations to correlate with prevalent atherosclerotic cardiovascular disease risk factors and with incident cardiovascular events, most commonly myocardial infarction [10, 99, 100].

Aggressive treatment to reduce traditional risk factors should be encouraged in SLE patients, including treatment of : 1. hypertension (goal: BP <130/85 mmHg), 2. hyperglycemia, 3.smoking cessation, 4. weight management with ideal body weight under 25 kg/m2 and 4. maintain regular physical activity [10]. Furthermore hyperlipidemia should be aggressively treated with an ideal LDL around 100 mg/dl. Statins are particularly attractive in treating hyperlipidemia because of their additional independent impact on lowering levels of C-reactive protein, at least in patients, with documented coronary artery disease [10, 101]. The spectrum of direct antiatherogenic properties of statins includes maintenance of endothelial function, anti-inflammatory actions, and a permissive action on smooth muscle cell proliferation that allows for synthesis of extracellular matrix proteins involved in the reparative response [10].Antimalarials may also be used because of its beneficial effects on lipid profile [102].

## CONCLUSIONS

Parallels between the inflammatory and immunemediated mechanisms of both atherogenesis and SLE may provide clues to understanding premature vascular disease in SLE patients. SLE-related factors are likely involved in all stages of atherogenesis from formation of the atherosclerotic lesion to its rupture, as well as in the thrombotic event itself.

SLE patients should be considered as high risk patients for CAD and modification of risk factors should be aggressively persuade in SLE patients.

## Figures and Tables

**Table 1:** CAD Risk Factors in SLE

Traditional risk factors	Immunological risk factors	Disease associated risk factors
Hiperlipidaemia	Immune complex damage	Corticosteroid use
Diabetes mellitus	Anntiphospholipid antibodies	Elevated homocystein levels
Smoking	Pro-inflammatory cytocines	Renal disease
Obesity		Hormonal
Hypertension		
Family history of CAD		
Sedentary lifestyle		
